# Fatal Coronary and Mesenteric Vasculopathy With Immune Checkpoint Inhibitor Therapy

**DOI:** 10.1016/j.jaccao.2026.09.008

**Published:** 2026-10-20

**Authors:** Aleksandra Abrahamowicz, Roberta Florido, Drew Ferguson, Fred Clayton, Monica P. Revelo, Anees Daud

**Affiliations:** a Division of Cardiovascular Medicine, University of Utah, Salt Lake City, Utah, USA; b Department of Pathology, University of Utah, Salt Lake City, Utah, USA

**Keywords:** acute coronary syndrome, cardiomyopathy, diagnosis, immunotherapy, ischemic disease, thrombosis

Immune checkpoint inhibitor (ICI) therapy has revolutionized the care of cancer patients across a broad range of malignancies. Although ICIs are generally well tolerated, they can cause immune-related adverse events (irAEs) that range from mild to life-threatening. Cardiovascular irAEs can include myocarditis, pericarditis, vasculitis, and arrhythmias.^1^^,^^2^ ICI-associated myocarditis has received significant attention because initial reported fatality rates ranged between 30% and 50%.^1^ However, patients receiving ICI therapy may also have an increased risk of non-inflammatory heart failure and coronary artery events through unclear mechanisms.^3^, ^4^, ^5^ Here we report the case of a patient with metastatic melanoma treated with ICI therapy who developed multiorgan ischemia and cardiogenic shock and ultimately died. Autopsy demonstrated a relative paucity of inflammation but highlighted a non-atherosclerotic vasculopathy characterized by intimal proliferation and luminal stenosis, ultimately causing ischemia, thrombosis, and organ failure.

This is the case of a 48-year-old man with history of metastatic melanoma for more than 5 years, multiple immune-related endocrinopathies and hepatitis, bipolar disorder, and remote methamphetamine and opioid use disorder in remission. His metastatic melanoma treatment history (Figure 1) initially included 13 cycles of pembrolizumab and was complicated by immune-related hypothyroidism. He then received dabrafenib and trametinib, which were transiently held for mildly reduced left ventricular ejection fraction (LVEF) which subsequently recovered. After disease progression, he was started on dual ICI therapy with ipilimumab and nivolumab; this was discontinued after 2 cycles for grade 2 hepatitis treated with steroids. He subsequently started single-agent nivolumab and received 3 doses. A computed tomography scan of the abdomen/pelvis obtained 3 weeks after the last infusion raised a concern for vasculitis given new diffuse circumferential arterial wall thickening and enhancement of the abdominal aorta and other branch vessels. His metastatic disease was stable. ICI therapy was discontinued and oral steroids were initiated.

**Figure 1:**
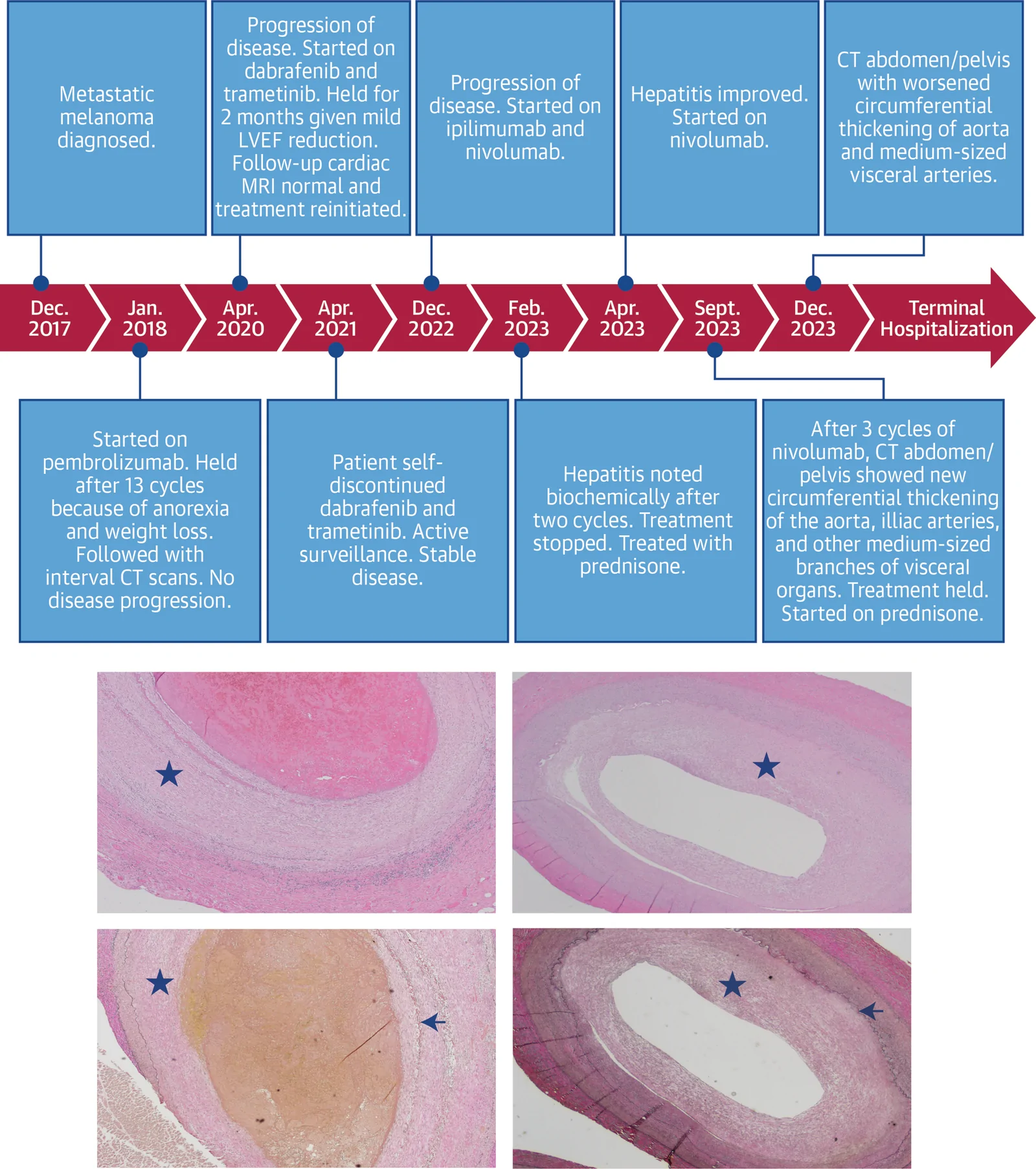
Clinical Timeline, Treatment Course, and Postmortem Histopathologic Findings Post-mortem autopsy slides are shown below the timeline. The first row of slides shows hematoxylin and eosin (H&E)–stained sections of coronary and inferior mesenteric artery, respectively. These show intima thickening (star) with a paucity of inflammation in the intima or media. In the case of the coronary artery section, there is luminal occlusion by a recent thrombus. The next row shows elastic stain of the coronary and inferior mesenteric artery, respectively. These highlight the preserved elastic lamina (arrow) and marked intima fibroblastic proliferation (star). LVEF = left ventricular ejection fraction; MRI = magnetic resonance imaging.

Approximately 4 months after the last dose of nivolumab, the patient presented to the emergency department reporting 2 months of progressive chest and abdominal pain, nausea, and dyspnea. He was alert and conversant but in significant discomfort. Blood pressure was 124/94 mm Hg and pulse 107 beats/min. Initial laboratory results were significant for lactate of 3.9 mmol/L, aspartate aminotransferase of 2,246 U/L, alanine aminotransferase of 1,659 U/L, total creatine kinase of 1857 U/L (reference, 20-200 U/L), B-type natriuretic peptide of 950 pg/mL, and troponin-I of 19.5 ng/mL (reference, 0-0.03 ng/mL). Erythrocyte sedimentation rate was 59 mm/h (reference, <15 mm/h). Electrocardiogram showed sinus tachycardia with inferolateral ST-segment depressions and T wave inversion. Echocardiogram showed severely reduced LVEF of approximately 15% with global hypokinesis and lateral wall akinesis. The differential diagnosis at this time included acute coronary syndrome (ACS), myocarditis, vasculitis, pulmonary embolism, and decompensated heart failure from an unrelated etiology.

CT angiography of the chest, abdomen, and pelvis showed diffuse wall thickening and enhancement of all visualized large- and medium-sized abdominal arteries with associated severe stenosis of the left gastric artery origin, severe stenosis of the superior mesenteric artery origin with focal distal occlusion, and severe stenosis of the superior renal artery.

He was initiated on intravenous methylprednisolone 1 g daily; however, he rapidly deteriorated and developed cardiogenic shock requiring intravenous inotropes. Troponin levels rose and peaked at 47.52 ng/mL. Despite these interventions, on hospital day 5 he developed severe abdominal pain with rising lactate. Imaging showed new splenic infarcts and possible bowel infarction. He was emergently taken to the operating room for an exploratory laparotomy, which showed severe bowel ischemia and required 100-cm small bowel resection. After a brief period of stability, on hospital day 8, he became acutely hypotensive, bradycardic, and pulseless. Resuscitative efforts were unsuccessful. The medical team and family opted to pursue an autopsy for further diagnostic clarity.

During his hospitalization, a broad differential diagnosis was explored. Given his abdominal pain and progressive arterial wall thickening involving multiple abdominal visceral organs, vasculitis was a major concern, and the rheumatology department was consulted. Serologic testing for antineutrophil cytoplasmic antibody–associated vasculitis was negative. Immunoglobulin G4 (IgG4)–related disease was unlikely given distribution of vessels involved, absence of retroperitoneal fibrosis, and normal IgG4 levels. Infectious aortitis was considered unlikely as blood cultures were negative, as was testing for tuberculosis and syphilis. Involvement of muscular arteries can be seen in medium-vessel vasculitides such as polyarteritis nodosa and Kawasaki disease; however, the patient did not fit the typical demographic profile for either condition. Small-vessel vasculitis was considered unlikely given the absence of cutaneous manifestations, proteinuria or hematuria, and pulmonary parenchymal involvement. Cryoglobulinemic vasculitis was unlikely given the patient’s hepatitis C viral load was undetectable and serum cryoglobulins were absent. Taken together, these findings supported ICI-related vasculitis as a unifying diagnosis to explain the cardiac and abdominal visceral organ ischemia. Concurrent myocarditis was also plausible, and coronary angiography, endomyocardial biopsy, and cardiac magnetic resonance imaging were all considered. However, the patient initially declined invasive evaluation and was deemed too clinically unstable to undergo prolonged diagnostic imaging. He was therefore empirically treated with high-dose corticosteroids to treat the suspected diagnoses. Although the patient had a history of methamphetamine use and use of dabrafenib and trametinib, both of which have been associated with left ventricular dysfunction and heart failure, these exposures were remote and would not explain either the multiorgan involvement or acute presentation.

Autopsy (Figure 1) demonstrated diffuse arterial wall thickening of medium-sized arteries, including coronary, splenic, hepatic, renal, superior and inferior mesenteric arteries. Notably, no atherosclerotic changes were present in these involved territories. There was extensive intimal fibromyxoid thickening, with marked vessel stenosis and patchy mild chronic inflammation. The intimal thickening was composed of a fibroblastic proliferation in a myxoid stroma. The samples demonstrated the presence of abundant acid mucopolysaccharides by alcian blue and colloidal iron special stains as well as minimal fibrosis by trichrome special stain. There was minimal lymphocytic infiltrate, perhaps because of several months of corticosteroid exposure. Immunohistochemical characterization was performed and demonstrated that most of the lymphocytes were T cells (CD3+) rather than B cells (CD20+). Many of them were CD8+ and thus likely cytotoxic T cells. Additionally, there was an occlusive thrombus of the left circumflex artery with adjacent areas of acute myocardial necrosis suggesting this to be the most proximal cause of death. The arterial findings resemble those observed in cardiac allograft vasculopathy.^6^^,^^7^

## Case Discussion

ICI therapy is an invaluable treatment option for several cancers, including those at advanced stages. Currently approved agents target immune checkpoints such as programmed cell death protein 1 (PD-1), programmed cell death protein-ligand 1, cytotoxic T lymphocyte-associated antigen 4, and lymphocyte-activation gene 3.^8^ Use of ICI has been hindered by irAEs that may occur in up to 70% of patients, resulting from unopposed activation of T-lymphocytes causing autoimmunity. ICI toxicity can affect most organ systems, and ranges from mild asymptomatic laboratory abnormalities to severe life-threatening conditions.

Pharmacovigilance analysis and observational studies have demonstrated an association between ICI use and ACS. Preclinical studies indicate that ICI therapies may accelerate progression of atherosclerosis, which has been proposed as the mechanism underlying the excess cardiovascular disease risk in these patients. Vascular inflammation has also been proposed as a potential mechanism leading to ACS. ICIs have also been implicated in the development of diffuse and localized vasculitis of small-, medium-, and large-sized arteries. Although vasculitis as an irAE is thought to be rare (<1% of patients treated with ICIs), a recent retrospective analysis indicated a fatality rate of 6.1%.^1^^,^^9^ Although the exact mechanism of irAEs remains unclear, it is suspected that upregulated T-cell activity combined with increased levels of proinflammatory cytokines and enhanced complement-mediated immune activity lead to an exacerbated inflammatory reaction.^8^ Most reported cases of ICI-associated vasculitis involve large-sized vessels and vessels of the nervous system during therapy with PD-1 inhibitors. This aligns with literature indicating a role of PD-1 in the pathogenesis of vasculitis whereby deficiencies in PD-1 lead to increased T-cell aggregation and cytokine production that leads to arterial wall inflammation. Few reports of ICI vasculitis include histopathology, given limited access to myocardial and vascular tissue. Those with available pathology generally include skin or kidney biopsies, and few are consistent with the 2022 American College of Rheumatology/European League Against Rheumatism Vasculitis Classification Criteria.^8^ Although there was a relative paucity of T lymphocytes in this patient’s tissue specimens, this finding may have been due to the patient’s prolonged exposure to steroids in the months before his presentation.

To our knowledge this is the first report with comprehensive histopathological analysis of diffuse coronary and abdominal visceral arterial involvement from an irAE. Additionally, our case is unique in demonstrating a paucity of vascular inflammation or atherosclerosis, and instead shows a diffuse arterial intimal proliferative vasculopathy leading to severe luminal stenosis and ischemia. This novel vasculopathy draws comparison to cardiac allograft vasculopathy (CAV) described in heart transplant recipients. Although not entirely understood, CAV is marked by diffuse, progressive coronary vessel wall thickening without atherosclerosis.^6^ CAV is thought to be a multifactorial process but on histology appears as an inflammatory fibroproliferative disorder involving predominantly the intima and has a component of T-cell–mediated inflammation.^7^

In this case, we postulate that the early imaging findings of vascular wall thickening were indicative of an initial inflammation process representing an irAE from ICI therapy, which may have led to progressive vascular narrowing through fibroproliferative remodeling, ultimately resulting in ischemic injury to both cardiac and abdominal visceral organs. This aligns with preclinical studies that show that an initial T-cell–mediated inflammation can ultimately trigger a vascular remodeling process with intimal proliferation and vessel occlusion.^10^ These findings should encourage additional study into the myocardial and vascular changes with ICI therapy and associated treatment implications. Although steroids are the current mainstay of therapy for irAEs, including vasculitis, they may only address an initial inflammatory response but not halt the secondary remodeling process.

In summary, we describe a case of multisystem vasculopathy most notably affecting the coronary and abdominal arteries, ultimately leading to diffuse cardiac and gut ischemia, cardiogenic shock, and patient death. On histopathology analysis, there was diffuse fibroproliferative arterial wall thickening and luminal stenosis, which may have resulted from an initial inflammatory trigger in the context of ICI therapy, and was unresponsive to steroid therapy. This case highlights 2 important considerations: 1) tissue-level data in cardiovascular irAEs are essential to enhance biological understanding, and 2) better phenotyping of ICI-related cardiac and vascular toxicities may allow for better diagnostic pathways and treatment options.

## Funding Support and Author Disclosures

The authors have reported that they have no relationships relevant to the contents of this paper to disclose.
